# Subjective experiences of tertiary student pianists with playing-related musculoskeletal disorder: a transcendental phenomenological analysis

**DOI:** 10.3389/fpsyg.2024.1303046

**Published:** 2024-04-23

**Authors:** Miao Xiaoyu, Ahmad Faudzi Musib, Indra V. Selvarajah

**Affiliations:** Faculty of Human Ecology, Universiti Putra Malaysia, Selangor, Malaysia

**Keywords:** playing-related musculoskeletal disorder, tertiary student pianist, experience, China, subjective

## Abstract

**Background:**

The literature suggests that the medical community needs musicians to provide an insider’s perspective to understand the physical and psychological dimensions of playing an instrument, and healthcare providers need to understand musicians’ experiences in order to develop coping strategies. Compared with professional pianists, student pianists are a neglected group. However, student and professional pianists both want to maintain their playing careers and have the experience of giving up playing because of playing-related musculoskeletal disorder (PRMD). There are a few studies conducted on student pianists’ experiences with PRMD, but none have been conducted in the Chinese context. Given the distinctive characteristics of higher music education in China and Chinese piano students, this study aims to investigate the lived experiences of tertiary student pianists with PRMD.

**Methods:**

Phenomenology is the most suitable qualitative method for investigating lived experiences. This study employed a transcendental phenomenological approach to investigate the experiences of student pianists, collecting data through one-on-one interviews and focus group discussions. Since phenomenological research emphasizes the homogeneity of research subjects, all 25 participants in this study are tertiary student pianists from seven Chinese higher education institutions.

**Results:**

Four themes and ten sub-themes were identified in this study. They are as follows: Theme one, Perceptions of PRMD, with sub-themes of body perceptions, negative thought, and emotional changes; Theme two, Complex Identity, with sub-themes of future pianists’ identity, nuanced identity of student pianists, and the dual identity between student pianist and patient; Theme three, Coping Strategies, with sub-themes of self-regulation and actively seek help from social relations; Theme four, Influences and Meanings, with sub-themes of negative influences of PRMD and positive meanings of PRMD.

**Conclusion:**

This study explores the experiences of tertiary student pianists with PRMD, including their subjective thoughts and feelings. It also highlights the importance of understanding tertiary student pianists’ experiences in developing health education and healthcare measures tailored to them.

## 1 Introduction

Musicians have high career satisfaction; however, amidst the happiness of playing music, there is also the risk of injury due to musculoskeletal disorders (Rose et al., 2021; Rodríguez-Gude et al., 2022). Playing-related musculoskeletal disorder (PRMD) is a common occupational disease among musicians (Cruder et al., 2020; Shinde and Borkar, 2021), but it has been neglected for a long time (Lee et al., 2012). The inaugural documentation of musician injuries in connection with their profession dates back to 1713, recorded in Ramazzini’s “Diseases of Workers” (Armocida, 2019). Subsequently, in the late 19th century, the British Medical Journal reported on musician injuries (Baxter, 2022). Notably, in the 1980s, The New York Times highlighted hand injuries experienced by pianists Gary Grafman and Leon Fleisher, with Grafman encountering difficulty in using his right hand and Fleisher diagnosed with focal dystonia, resulting in the loss of his ability to play the piano with his right hand. Both pianists shared their experiences of playing injuries through the media, bringing widespread public attention to musicians’ health problems for the first time (Dunning, 1981).

For an extended period, there was a lack of precise terminology to characterize musicians’ playing-related health issues. Expressions like overuse syndrome, repetitive strain injury, and misuse were frequently employed to delineate injuries associated with playing (Zaza, 1998). Zaza et al. (1998) conducted a literature review on musculoskeletal disorders in musicians and introduced the term play-related musculoskeletal disorders (PRMD) to encompass such conditions. PRMD is defined as “pain, weakness, numbness, tingling, or other symptoms that interfere with the ability to play the instrument at the level one is accustomed to.” This definition by Zaza et al. (1998) currently stands as the most widely accepted and employed terminology in the field (Shanoff et al., 2019).

The risk factors and symptoms of PRMD vary among musicians playing different instruments: for instance, tenosynovitis in the hands of keyboard players, nerve problems in the upper limbs and backs of percussionists, and cervical spondylosis, periarthritis of the shoulder, and back musculoskeletal diseases in orchestral musicians. Wind players are prone to oral and lung diseases (Bronwen et al., 2012; Larsen, 2020; Mizrahi, 2020). Most existing literature focuses on orchestra musicians (Rotter et al., 2020; Ryan et al., 2021), though independent musicians are also susceptible to PRMD, with varying experiences due to different musical instruments. Hence, it’s imperative to study homogeneous groups of musicians playing the same musical instrument for more in-depth and representative research conclusions (Panebianco, 2021; Svendsen, 2022). This study is conducted in the context of China, where piano students account for the largest proportion of professional or amateur music education, and piano is also the preferred instrument for students to learn (Yong et al., 2019). Therefore, this study chooses Chinese student pianists as the research object, which is more representative.

Current literature indicates a prevalence of PRMD ranging from 62 to 93% among professional musicians (Smyth and Mirka, 2021) and 26 to 93% for pianists specifically (Bragge et al., 2006). However, PRMD is not exclusive to professionals; student musicians, particularly at the tertiary level, also grapple with this challenge. Among conservatory students, the prevalence of PRMD varies from 43 to 63% (Stanhope et al., 2019; Cruder et al., 2020; Portnoy et al., 2022). A study on the occupational health course experiences of tertiary music students revealed that 75% of student musicians had encountered PRMD (Salonen, 2018). Brandfonbrener (2009) identified an 85% incidence of PRMD among first-year music students, with some affected even before entering college. Certain studies propose that student musicians face a greater risk of PRMD than their professional counterparts due to limited playing experience and injury-coping strategies (Cruder et al., 2020). The impact of PRMD on student musicians may result in reduced learning efficiency or discontinuation of music education (Kok et al., 2016; Steemers et al., 2020). Despite these implications, the injuries and experiences of student musicians often go unnoticed, rendering them a frequently overlooked group (Détári and Nilssen, 2022; Matei and Ginsborg, 2022).

With the progress of performing arts medicine, an increasing number of studies are honing in on PRMD. However, the predominant focus in current literature remains quantitative, with prevalence and risk factors being frequently explored (Kaufman-Cohen and Ratzon, 2011). Quantitative research, while valuable, may not capture the nuanced aspects of pianists’ experiences or unveil the specificity and social factors in their experiences with PRMD (Queirós et al., 2017; Cruder et al., 2020). Hence, the inclusion of qualitative research becomes imperative. Qualitative studies play a crucial role in aiding clinicians to formulate effective treatment plans for pianists, identifying research gaps, and informing future studies on PRMD-related subjects (Etchison and Kleist, 2000). According to Guptill and Golem (2008), practitioners’ anecdotes on successful coping with PRMD constitute valuable contributions to the literature on musicians’ health issues. Building on this, Guptill (2010) emphasizes that treatments offered to musicians are most effective when healthcare providers comprehend the nature of the challenges they face. Therefore, this study focuses on the lived experiences of student pianists.

### 1.1 The experiences of musicians and student pianists related to PRMD

Previous research shows that musicians generally are highly satisfied with their careers and believe that work provides them with identity and self-worth. This conviction enables them to persevere in their careers despite experiencing PRMD and find fulfillment in their musical pursuits (Park et al., 2007; Guptill, 2010; Hale, 2019). Interestingly, a study identified a paradoxical relationship between the happiness derived from performing music and the presence of PRMD among musicians. They reported feeling content rather than distressed when striving to sustain their playing careers post-injury. However, this happiness may inadvertently lead them to willingly continue working in a state of physical discomfort, thereby heightening the risk of PRMD (School and Zosso, 2012). After suffering from PRMD, musicians commonly undergo a sense of “loss of prior ability” and diminishing dignity due to impaired performance (Pappa, 2019).

The literature on PRMD is often focused on professional pianists, so the lived experiences of injured student pianists are often overlooked (Austen, 2020). However, student pianists identify with their profession as highly as professional musicians do and are willing to play despite the high risk of injury (Park et al., 2007). Student musicians also enjoy the process of creating and performing music, so they face a dilemma between the “need to play” and “respect the body” after undergoing PRMD (McCready and Reid, 2007). They are also prone to emotional distress and career uncertainty (Cruder et al., 2020). Bragge et al. (2006) investigated pianists’ perceptions of PRMD, as well as their behavioral, emotional, and physical worlds through interviews. The results show that pianists are more likely to suffer from PRMD than other instrumentalists, and they also experience many internal and external stresses. Pianists are reluctant to declare their health problems, which causes therapists to lack understanding of their experiences and needs, so the effect of PRMD management accepted by them is not ideal. In addition, due to the lack of PRMD prevention courses in higher education institutions, student pianists lack knowledge and awareness of PRMD (Ling et al., 2018). Waters (2020) proposed that student pianists have a low sense of self-efficacy, are prone to stress and anxiety, and lose control of life, so student pianists need to develop time management skills to maintain the balance between physical and mental health and piano performance. Some PRMD-related studies also have found that social relationships significantly impact student pianists with PRMD. Lack of support from interpersonal relationships such as family, teachers, and classmates can lead to psychological stress, emotional distress, and physical suffering (Santos and Queirós, 2019). The existing literature can prove that qualitative studies can help to understand pianists’ thoughts, feelings, behaviors, interpersonal relationships and other hard-to-quantify experiences that are important for coping with PRMD and sustaining their playing careers. The experiences of Chinese tertiary student pianists with PRMD have not been investigated, so this study focuses on Chinese student pianists.

### 1.2 Transcendental phenomenology

Moustakas (1994) created transcendental phenomenology based on the theories of Husserl and Heidegger. The distinction between transcendental phenomenology and other phenomenological approaches is that it proposes that the research process leads researchers back to the potential meaning of experience, and the main task of phenomenological researchers is to describe what is presented in the data and what may be hoped or imagined. Emphasis is placed on the interpretation of underlying existential dynamics through intuition, imagination, and universal structures. These dynamics can bring about aspects of personal experience (sensations, thoughts, and perceptions) into consciousness (Moustakas, 1994). Moustakas developed two main types of data analysis: textural and structural. Textural analysis is used to analyze the experiences that appear in participants’ consciousness and describes participants’ perceptions of a phenomenon (Yüksel and Yıldırım, 2015). Structural analysis is used to reveal implicit universal structures, which are common characteristic markers of the lived experiences of people who have experienced the same phenomenon, and to describe how the phenomenon is experienced by people (Lopez and Willis, 2004). Textural analysis and structural analysis are complementary, just as human experience and how a phenomenon is experienced cannot be separated (Ihde, 2012).

### 1.3 The application of transcendental phenomenology to musicians’ health problems

Phenomenological studies focusing on musicians’ health issues predominantly employ the research method of interpretive phenomenology (IPA) (Salonen, 2018; Hale, 2019; Casey, 2020), with a more limited application of transcendental phenomenology. Howard (2004) stands as a pioneer in utilizing transcendental phenomenology to explore musicians’ health problems. Her study examined the experiences of musicians who lost their ability to play due to overuse injuries, revealing a cyclic process from loss to recovery, ultimately leading musicians to establish a new balance. Noteworthy contributions from Burgoyne (2022) and Bober (2019) delve into musicians’ lived experiences concerning perfectionism and performance anxiety, underscoring the importance of recognizing psychological issues in musicians. In a different vein, Olson Moser (2021) explores the lived experiences of professional musicians grappling with focal task-specific dystonia, offering insights from a musician’s perspective.

### 1.4 Social cognition theory (SCT)

Social cognition theory (SCT) is a theory about individual behavior created by Bandura. The core idea is ternary interaction determinism, that is, there is a dynamic interaction between individual cognition, environment, and behavior (Bandura, 1986, 2002). Bandura (1986) argued that people can take control of their lives by increasing self-efficacy and outcome expectations and becoming self-regulators. Although there is no literature on the use of SCT to study musicians’ health problems, SCT is often used in phenomenological research, such as Gallagher et al. (2005) studying the contribution of phenomenology to SCT and Rashid (2022) using SCT to investigate the lived experiences of social media influencers related to cyberbullying. SCT is also a common theoretical model in the field of health behavior research (Bandura, 2002; Painter et al., 2008). SCT guides this study to investigate tertiary student pianists’ PRMD-related experiences from the perspectives of personal, behavior, and environment factors and to develop a deeper understanding of their experiences by using the two SCT characteristics of self-efficacy and outcome expectations. Therefore, the innovative use of SCT in this study is feasible.

### 1.5 Current study

In China, researchers commonly characterize health issues related to instrumental music as “musicians’ occupational diseases” (Li, 2006; Wang, 2008; Tang, 2010). Despite this terminology, there is a noticeable dearth of research on playing-related Musculoskeletal Disorders (PRMD) in China, particularly concerning student pianists. The health concerns of student pianists have not received sufficient attention, and higher education institutions in China lack dedicated facilities for managing and treating PRMD in music students. Moreover, there is a notable deficiency in health education initiatives tailored to the needs of music students. Although qualitative research is difficult to generalize to a wider group, this preliminary study in China serves as a valuable starting point, aiming to draw attention to the health problems of student musicians and their associated experiences, with the hope of inspiring broader awareness and further research efforts.

This study is designed on the basis of transcendental phenomenology, with Social Cognition Theory (SCT) as the theoretical perspective. It focuses on the experiences of tertiary student pianists related to PRMD, and delves into their thoughts and feelings, emphasizing the descriptions of participants’ lived experiences (Langdridge, 2007). In light of the lack of research on the experiences of student pianists and the absence of an Asian perspective in the PRMD research field, this study aims to explore the lived experiences with PRMD from injured Eastern tertiary student pianists’ perspective so as to better understand their thoughts, perspectives, and feelings. This understanding can, in turn, encourage the healthcare and education sectors to pay attention to the ideas and needs of student pianists, thereby providing them with appropriate help and support. The research questions of this study are as follows:

1. What are the lived experiences of tertiary student pianists related to PRMD?
2. What are the subjective thoughts and feelings of tertiary student pianists who have suffered from PRMD?

## 2 Materials and methods

### 2.1 Study design

This study employs a phenomenological design, focusing on the experiences of tertiary student pianists afflicted with PRMD, utilizing a transcendental phenomenological approach for analyzing these experiences (Moustakas, 1994). Moustakas (1994) posited that investigating the common experiences of several individuals concerning a specific phenomenon is the most suitable type of research problem for phenomenology. Transcendental phenomenology focuses on the structures of consciousness and the ways in which they shape human experience rather than on interpreting the experiences of participants and the meaning of those experiences (Moustakas, 1994). It is consistent with the aim of this study to understand participants’ thoughts, feelings, perspectives, and behaviors through in-depth descriptions of their lived experiences related to PRMD without the investigator’s interpretation of their experiences.

### 2.2 Participants and recruitment procedure

Kaufman-Cohen and Ratzon (2011) proposed that purposive sampling is the best method when research requires collecting information from interviewees based on their attributes or characteristics. This study required assurance that each participant was a student pianist at a higher education institution, had prior experience with PRMD, and met the recruitment criteria for the study. Therefore, the purposive sampling method was adopted to recruit participants for this study. The recruitment criteria are as follows: (1) student pianists from higher education institutions with experience related to PRMD and pain intensity of at least level 3 [self-assessed according to Fry’s (1986) method]; (2) have significant experience related to playing the piano and PRMD; (3) be willing to discuss their injury experiences.

Upon obtaining ethical approval from the Ethics Committee for Research Involving Human Subjects at the university where the researcher was affiliated, the researcher initially contacted 42 potential interviewees through the recommendation of piano teachers from six comprehensive universities and one conservatory in China, introduced the purpose of the study and its research significance to them, and inquired about their willingness to participate in the interview. Upon discussion, the researcher discovered that some of the student pianists suffered musculoskeletal disorders due to playing a second instrument (an instrument other than the piano), engaging in sports, or other causes. Some individuals were unable to communicate openly due to their status as researchers and teachers or other reasons, which was not conducive to data collection. Others declined to be interviewed for fear that their personal information or injury experiences would be exposed. Ultimately, after excluding student pianists who did not meet the recruitment criteria and did not wish to be interviewed, 25 student pianists participated in the study, of which 12 engaged in one-on-one interviews, and 13 partook in focus group discussions across two groups. One-on-one interviews serve as a valuable means to gain insight into individual participants’ experiences and thoughts regarding playing-related Musculoskeletal Disorders (PRMD). On the other hand, focus groups offer the opportunity to comprehend the collective experiences of a group, providing a broader perspective and potential confirmation of individual participants’ experiences. The synergistic use of both methods ensures a more comprehensive understanding of participants’ subjective experiences, capturing both the nuances of individual participants and the shared perspectives within the focus group. The basic information regarding the participants is depicted in Tables 1, 2.

**Table 1:** Demographic information of one-on-one interview participants.

	Variable	Range	Percentage
Age (years)	Minimum	17	/	Maximum	26	/	Median	22	/
	Gender	Male	5	41.7	Female	7	58.3
		Educational background	Bachelor	8	66.7	Master	4	33.3
Piano learning years	Less than 1 year		0	0	2–5 years	2	16.7	6–10 years	6	50.0	More than 10 years	4	33.3
	PRMD severity	No PRMD (below level 3)	0	0	Moderate PRMD (level 3–5)	9	75.0	Severe PRMD (level 6–7)	3	25.0

**Table 2:** Demographic information of focus group discussion participants.

	Variable	Range	Percentage
Age (years)	Minimum	18	/	Maximum	28	/	Median	21	/
	Gender	Male	5	38.5	Female	8	61.5
		Educational background	Bachelor	9	69.2	Master	4	30.8
Piano learning years	Less than 1 year		0	0	2–5 years	5	38.5	6–10 years	5	38.5	More than 10 years	3	23.1
	PRMD severity	No PRMD (below level 3)	0	0	Moderate PRMD (level 3–5)	10	76.9	Severe PRMD (level 6–7)	3	23.1

### 2.3 Data collection

Qualitative research is conducted through one-on-one communication with individuals or direct interaction within a group to obtain in-depth information and understanding of the subject (Bernard, 2013). Individual face-to-face interviews and focus groups are the most suitable data collection methods for phenomenological research (Shorey and Ng, 2022). Data were collected in the form of one-on-one interviews and focus group discussions, both of which were conducted in a semi-structured manner. Regular questions were prepared in advance as interview guides (see Appendix 1) to elicit unstructured responses. To allow participants to accurately and fully describe their experiences, questions could be added, replaced, or removed during the interview (McIntosh and Morse, 2015). Prior to the formal data collection, researchers initiated one-on-one pilot interviews with five tertiary student pianists who were not part of the main participant group. Subsequently, pilot group discussions were conducted with these same five students, leading to minor adjustments in the interview guidelines based on the feedback received. The results of the pilot study indicated that one-on-one interviews were more conducive to obtaining in-depth, detailed, and personal data. Focus group discussions proved effective in yielding broader information, and participants demonstrated a greater likelihood of opening up during interactive sessions.

Before the formal interview and discussion, the researcher distributed informed consent forms to the participants, providing the overview of the study’s purpose and outlining the data collection procedures. Participants who signed and returned the informed consent forms were considered to have given their consent to participate and retained the right to terminate or withdraw from the interview at any point. Throughout the process, the researcher ensured the anonymity and confidentiality of the participants. In the one-on-one interviews, participants were identified as Pianist 1, Pianist 2, and so forth, up to Pianist 12. The group representing conservatory student pianists was denoted as Focus Group 1, consisting of six individuals, while the group representing non-conservatory students was labeled Focus Group 2, comprising seven individuals. With the participants’ consent, the interviews and discussions were recorded and securely stored in an encrypted folder on the researcher’s computer. The recordings would be deleted upon the conclusion of the study, and all data were used exclusively for research purposes (Chen et al., 2018).

One-on-one interviews preferred face-to-face format. Due to the rehearsal, treatment, and concert preparation, the three participants were still unable to arrange the time and place of the face-to-face interview after many times of communication. Hence, they used the form of the telephone interview, and the total time of the interviews was about 25–40 min. Focus group discussions were conducted face-to-face and lasted 67 min (Focus Group 1) and 73 min (Focus Group 2). Participants had the freedom to choose interview settings. One-on-one interviews took place in varied locations such as coffee shops, dessert shops, the library, and the piano room. The two focus groups convened in university classrooms. Each individual participant and focus group contributed to a single interview session, resulting in the collection of 14 interview datasets. The interviews and discussions involved the lived experiences of tertiary student pianists with PRMD. Data collection was stopped when all the themes emerged and strongly supported the research questions, and participants felt they had fully shared their experiences. The data reached saturation when the eleventh participant was interviewed, and the focus group discussion further validated and supported the interview data.

### 2.4 Data analysis

Data were analyzed using Moustakas’ refined Stevick-Colaizzi-Keen (SCK) phenomenological method (Moustakas, 1994; Morrow et al., 2015) and the data analysis software Nvivo for the coding process (QSR International Pty Ltd., 2020). Table 3 delineates the steps in the Stevick-Colaizzi-Keen (SCK) method.

**Table 3:** Analytic steps of Stevick-Colaizzi-Keen (SCK).

Steps	Concrete content
1	Familiarising
2	Identifying significant statements
3	Converting significant statements into formulated meanings
4	Categorise meanings to develop themes
5	Exhaustive description
6	Producing the fundamental
7	Seeking verification for validation of findings

Initially, the researcher transcribed the verbatim recordings of one-on-one interviews and focus group discussions into documents to ensure complete records of the participants’ experiences. The researcher then scrutinized the transcripts repeatedly until she became fully acquainted with the participants’ experiences with PRMD, following which she categorized the data using open coding. Subsequently, specific meanings were drawn out from significant statements based on the text of the explanation. Each meaning derived from the significant statement was then amalgamated to develop themes related to the participants’ experiences. Redundant, irrelevant, and trivial descriptions were expunged from the overall structure to concentrate on the fundamental structure pertinent to the participants’ experiences. Finally, the transcript and coding stripes were forwarded to the participants to verify whether the researcher’s summarization and depiction of their experience were accurate and apt (Majabadi et al., 2016).

### 2.5 Credibility

In adherence to the tenets of phenomenological study, and to avert the researcher’s subjectivity from influencing the research results, the researcher continually reminded themselves during the research process to: (1) extricate themselves from the phenomena and underscore their role as “research tools”; (2) not to prefigure what the participants might describe, holding no anticipation regarding the outcome (Moran, 2002; Lopez and Willis, 2004); (3) avert the researcher’s own preconceived notions as a piano educator and a pianist (Lopez and Willis, 2004).

The processes of participant sampling, data collection, and data analysis in this study all adhere to the criteria of transcendental phenomenology (Moustakas, 1994; Thomas and Pollio, 2002; Boddy, 2016). Transcripts and analyses of the study were circulated back to participants to confirm and amend the results (Majabadi et al., 2016). Furthermore, the study was overseen by members of the supervisory committee (Merriam and Tisdell, 2015), and the second and third authors, serving as members of the supervisory committee of the first author, reviewed and monitored the entire research process, providing invaluable guidance and feedback for the study.

## 3 Results

This study endeavors to explore the experiences of tertiary student pianists with PRMD, focusing on their subjective thoughts and feelings. The research identifies four central themes: perceptions of PRMD, complex identity, coping strategies, and influences and meanings. Table 4 provides an overview of the primary themes, subthemes, and occurrences documented in this study.

**Table 4:** Emerging themes and sub-themes.

Themes	Sub-themes	Occurrence
Perceptions of PRMD	Body perceptions	9 interviewees 2 focus groups
	Negative thought	8 interviewees 2 focus groups	Emotional changes	9 interviewees 2 focus groups
	Complex identity	Future pianists’ identity	11 interviewees 2 focus groups
Nuanced identity of student pianists		7 interviewees 1 focus groups
The dual identity between student pianist and patient		5 interviewees 1 focus groups
Coping strategies	Self-regulation	10 interviewees 2 focus groups
	Actively seek help from social relations - Problem-focused coping strategies - Emotion-focused coping strategies	8 interviewees 2 focus groups
Influences and meanings	Negative influences of PRMD	11 interviewees 2 focus groups
	Positive meanings of PRMD	7 interviewees 2 focus groups

### 3.1 Perceptions of PRMD

Participants described their perceptions associated with PRMD as multifaceted. The most immediate sensations included physical pain and discomfort, restricted physical activity, and negative thoughts and emotional responses.

#### 3.1.1 Body perceptions

When participants were asked to recall when they discovered they were suffering from PRMD, most could only name a general period. They described this as “around the time of the college entrance exam,” “when I first entered the conservatory,” “when there was a final exam,” and “about a year ago.” Some participants had very vague or no recollection of PRMD at all. However, all participants could clearly remember the feeling of physical pain and discomfort, as described by a member of Focus Group 1:

"I do not remember the exact date, but about a year ago, I started having pain and swelling in my shoulders, lower back and neck. At the time, I did not think it was a big problem. I thought if I reduced my practice time, these feelings would go away. But last summer vacation, I spent two weeks in Beihai with my friends. During that time, my discomfort did not abate. I still feel pain and stiffness in my body every day." (Focus Group 1)

Pianist 5 mentioned that although she could not remember when the PRMD occurred and did not know she was suffering from periarthritis of the shoulder until the hospital diagnosed it, she could clearly remember the signs of injury in her body:

"At the time, my hand was very sore and sore when I lifted it up, and my arm also felt numb and swollen. In retrospect, in fact, during that time, I was able to be identified as having periarthritis of the shoulder, especially in the case of limited mobility of the shoulder joint, which was a very important signal." (Pianist 5)

The feeling of physical pain and discomfort persisted throughout the participants’ post-injury experience, not merely when suffering from PRMD. Participants reported experiencing physical discomfort while playing the piano, doing other tasks, or even doing nothing at all, and the pain even became more pronounced and severe over time:

"I am now quite helpless. I have noticed that the pain in my shoulder and arm seems to have become persistent. I now play the piano and write papers; even if nothing else, I would feel the pain and the stiffness of the shoulder. I feel like my health is getting worse." (Pianist 9)

#### 3.1.2 Negative thought

After suffering from PRMD, participants had negative thoughts and feelings. This change mainly manifests in losing confidence in their ability to play, feeling that their self-esteem has been hurt, and feeling that their career development path is limited. A few participants with severe cases even developed musical performance anxiety:

"Periarthritis of the shoulder really made me feel pain in my heart at one point, and I even wanted to leave this world. Suffering from this disease hurts my self-esteem very much. Before I was a strong person, I thought I was invincible. But now that I'm sick, I have no control over my body, and I feel very uncomfortable." (Pianist 5)

"When I learned that I was suffering from tenosynovitis, I felt that the human body was too fragile and that it limited my soul. My ideals cannot be realized, my dreams are trapped by my body." (Pianist 4)

"I suffer from performance anxiety because of PRMD, and I feel like I have lost the ability to play a piece in its entirety on stage. During concerts, exams, or competitions, I can not control my heart rate, my anxiety, my shaking legs and the sweat in my palms. I may not be able to become a professional Pianist after graduation." (Pianist 7)

#### 3.1.3 Emotional changes

Participants underwent a range of emotional changes after suffering from PRMD. However, the overarching trajectory was a progression from initial disbelief to eventual acceptance.

When the participants first learned that they had suffered from PRMD, they had difficulty believing and accepting that playing the piano had caused their injuries. They were also reluctant to accept the fact that they were injured. This disbelief was followed by feelings of sadness and frustration, as well as anxiety about future career development. Participants who tried various methods of pain relief and received professional treatment but did not improve felt helpless, hopeless, and even angry. However, after a longer period of PRMD, most participants began to accept their injuries and became more emotionally peaceful. Pianist 8 described his feelings of helplessness and despair:

"To be honest, I was an emotional wreck. I am so young, so I can not handle it. I felt very desperate, and I did not have big dreams. I just wanted to play the piano. So why is the world doing this to me? It is really hard for me to control my emotions. I am so helpless "(Pianist 8).

Pianist 4 presented a more comprehensive description of her emotional changes:

"When I first confirmed that I was injured, it was incredible. I was also worried that my ability to play would be affected. Then, there was anxiety and restlessness. My doctor told me I had to stop playing for a month. I was afraid I would not be able to play the piano again. But then, after a long period of therapy, my therapist told me that many pianists face the same problems as me, and I was relieved that I was not the one who suffered the most. Everyone else is playing, so I cannot give up." (Pianist 4)

### 3.2 Complex identity

The participants’ sense of their own identity is intricate and uncertain. While they take pride in their identity as pianists, they grapple with the doubts and uncertainties introduced by the identity of being student pianists. Concurrently, there’s an identity conflict between the pianist and the patient.

#### 3.2.1 Future pianists’ identity

Though participants possess tertiary student identities besides being pianists, they identify as pianists as fervently as professional pianists do. They perceive the pianist identity as an embodiment of their personal value, capabilities, and societal standing. As Pianist 4 remarked, “Without the title of a pianist, I’m unsure of who I am or how I’d present myself to others.” Being a pianist also mirrors the participants’ strengths and passions. Pianist 9 elucidated, “This identity assures me of my proficiency at the piano and my potential to turn my passion into a profession.” Moreover, several participants felt a deep sense of honor and pride in their status as pianists. They were of the belief that others would value and acknowledge them due to their pianist identity. This recognition would not only bring them joy but also motivate them toward a career in playing. A member of Focus Group 2 shared:

"I am proud to be a pianist. I feel that I can be appreciated by others, although I am so insignificant when I am not playing the piano, which is also my motivation to become a professional pianist. I am satisfied that being a pianist allows me to get everything I want from my spiritual dimension." (Focus Group 2)

#### 3.2.2 Nuanced identity of student pianists

A few participants drew a distinguishing line between “pianist” and “student pianist”—a differentiation that often exposes them to disregard, skepticism, and career uncertainties.

Pianist 10 highlighted how the well-being concerns of student pianists often go unheeded:

"Successful pianists have their own health management team… The average professional pianist also has access to good therapists. You see, like us as students, the university does not teach us how to deal with injuries, and the teachers just tell us to practice hard, no pain, no gain. All the physical impairments associated with playing the piano seem to be rationalized, and no one, including ourselves, will take our own body seriously anymore." (Pianist 10)

Pianists 6 and 11 discussed how student pianists’ abilities are readily doubted by the general public. Despite possessing remarkable skills, their expertise during public concerts, part-time teaching, or piano contests is often under scrutiny. Such skepticism intensifies when they are afflicted by PRMD. Pianist 12 narrated an instance when her condition became a subject of skepticism:

"Although I have given a lot of solo concerts, I know that some new audiences have doubts about my playing. After all, I am still a junior student. Especially after my injury, these doubts have increased, and I feel anxious and bitter about them." (Pianist 12)

Moreover, certain participants expressed that the identity of student pianists is fraught with uncertainty, and they cannot assure themselves of becoming professional pianists post-graduation. Suffering from PRMD increased this uncertainty, casting further doubt on whether their physical condition would sustain a long-lasting playing career. Pianist 9 described it in the following manner:

"I can not confidently tell people I am a pianist because I am still a student, and maybe no one will hire me after graduation, so I will say goodbye to the piano playing profession. Then, I got hurt again and was even more unsure…" (Pianist 9)

#### 3.2.3 The dual identity between student pianist and patient

A segment of participants conveyed that post-PRMD, they wrestled with a dual identity: being a pianist and simultaneously a musculoskeletal patient. This stark juxtaposition subjects them to immense identity conflict and emotional strain. Pianist 2 shared his sentiments:

"Being a pianist is good for me, and it gives me a social identity. But at the same time, the physical pain reminded me that I was a sick person. So I am a pianist, and I am a patient at the same time, and it is so conflicted. I feel embarrassed, and others will have a negative opinion of me." (Pianist 2)

### 3.3 Coping strategies

The coping strategies reported by the participants included self-regulation and help from social relationships. Self-regulation was embodied in participants’ spontaneous improvement of behaviours that may lead to injury, cultivation of behaviours conducive to the remission of PRMD, and self-psychological regulation. Help from social relationships was embodied in assisting participants in dealing with PRMD-related problems and providing emotional support.

#### 3.3.1 Self-regulation

The self-adjustment methods mentioned by participants include changing playing methods, adjusting living habits, and self-psychological adjustment. Most participants adjusted their playing skills, posture, and habits after the injury, primarily by improving the playing skills that may cause injury to the body, trying more ergonomic playing postures, and avoiding over-practising. The members of Focus Group 1 discussed the benefits of changing the playing method. One member mentioned:

"In the long-term learning of performance, we may neglect the correct way of playing because of the pursuit of beautiful timbre and superb technique. For example, if I play a grand work, in the past, I might overuse the power of my fingers in order to produce a strong note. Now, I think that the finger is only the fulcrum of the body's strength. As long as I pass the strength of the body smoothly to the fingertips, my fingers will not be so tired and so painful. The body should be one when playing the piano." (Focus Group 1)

Pianist 2 alleviated physical pain and discomfort by adjusting life habits:

"I think it is important for a pianist to have a good lifestyle. I now give myself time to rest during practice and usually pay more attention to rest and sleep. I have also started to do jogging and aerobic exercise every day to ease my discomfort from the point of view of improving my muscle capacity." (Pianist 2)

Finally, some participants found that self-regulation was also helpful. They would give themselves positive psychological suggestions before practising or playing the piano to adjust their psychology to a better state. For instance, Pianist 3 would tell himself before playing the piano, “I will definitely play this piece perfectly.” Some participants did meditation exercises to calm and relax their minds:

"Meditation reduces my psychological stress and anxiety. I would bring my consciousness back to my body. For example, I imagine that the pain in my body, especially my shoulders, is gone, and my body is light and warm. I do not know if it is a psychological effect, but every time I finish my meditation, I feel physically and psychologically relaxed, and I feel less pain." (Pianist 1)

#### 3.3.2 Actively seek help from social relations

The help that social relationships provide to participants is mainly embodied in substantive help (problem-focused coping strategies) and emotional support (emotion-focused coping strategies).

##### 3.3.2.1 Problem-focused coping strategies

Substantive, problem-solving-focused help is provided by piano teachers and therapists. Some of the participants’ piano teachers designed more scientific playing methods for them. They helped them choose pieces suitable for their physical conditions to reduce the possibility of injury due to playing the piano:

My teacher helped me adjust my playing skills after my injury. When choosing the repertoire, he no longer only let me play difficult works but also let me play a lot of difficult but emotionally powerful works. While the pain is relieved, I also get an improved sense of music. (Pianist 1)

In addition, all of the participants in the one-on-one interview had been treated by a healthcare professional, including TCM therapy, physical therapy, and medication:

"I have had triamcinolone injections, I have taken anti-inflammatory drugs. I also tried Chinese medicine treatment, acupuncture, massage and so on. I prefer to receive Chinese medicine treatment. I also had electrotherapy and massage. I also received acupuncture treatment from traditional Chinese medicine. I have tried everything I can think of." (Pianist 12)

##### 3.3.2.2 Emotion-focused coping strategies

The participants’ experiences demonstrate that while only their piano teachers and healthcare providers were able to provide them with substantive help, they had a wide range of social connections that could offer emotional support, including their families, teachers, friends, classmates, and even some of the more distant listeners, viewers, and competition judges.

Pianist 5 discussed the care, understanding and love she received from her family and teachers after suffering from PRMD:

"I suddenly found that my teacher was friendly to me, and he was not overly strict with me as before. Now, he cares more about my emotions and my health, makes me put my body first, and tells me there are other good things in life besides playing the piano. My parents also began to understand my situation and stopped pushing me. I know they love me, they just want me to have a good future." (Pianist 5)

Pianist 8 mentioned the company and support of his classmates and friends during his playing career and coping with PRMD:

"I am lucky that on the road to pursuing my dreams and during the time I spent healing my body, I made a group of like-minded friends. We exchange experiences, share our thoughts together, and strive to be better together. I am grateful to them." (Pianist 8)

Pianist 12 mentioned that the acceptance and understanding of concertgoers were what kept her going as a professional pianist:

"I was in a recital when my fingers began to spasm because of tenosynovitis during the climax, and all five fingers stuck together like glue, so I had to stop playing. However, as I awkwardly bowed to the audience, there was a burst of applause. I felt very surprised. Instead of blaming me, the audience forgave and encouraged me. It was so healing for me that it made me feel like I had to be a professional Pianist no matter what difficulties I faced." (Pianist 12)

### 3.4 Influences and meanings

Participants reported that their experiences with PRMD negatively impacted their performance, lives, bodies, and minds, but they also garnered some positive insights from them.

#### 3.4.1 Negative influences of PRMD

Participants conveyed that the most noticeable setbacks after suffering from PRMD were the deterioration in performance and the instability in their playing. These challenges not only imposed considerable psychological burdens and inconveniences but also posed obstacles to the progression of their future playing careers. Additionally, there was an increased likelihood of participants contemplating abandoning their playing careers. Pianist 1 mentioned that after suffering from PRMD, she could no longer play difficult and physically demanding works, even pieces that were previously manageable, and she could noticeably feel the decline in her playing ability:

"My body now cannot support me to play difficult piano pieces, such as Liszt's Etudes and Chopin's concertos. Even when I play pieces that I used to be able to do easily, I feel very difficult and physically uncomfortable. My playing dropped too much after the injury." (Pianist 1)

Pianist 11 expressed that suffering from PRMD affected his playing state, making it challenging for him to concentrate and immerse himself while playing, and the efficiency of his practice was also reduced:

"After the injury, getting into shape was hard for me. Physical pain distracts me and makes it difficult to calm down. Especially when the teacher assigned a new piece of music, I felt that my speed of mastering a piece of music was always slower than that of other students because I could not play it for a long time. I feel so anxious and under great psychological pressure." (Pianist 11)

Some participants described physical pain and discomfort as sources of distress and inconvenience in their lives, mainly as an inability to perform daily activities, housework, and physical exercise due to physical pain. Some participants experienced severe pain and discomfort in cold, wet weather. Participants with severe PRMD needed assistance even for basic daily activities:

"I feel that PRMD has seriously affected my daily life. When my lumbar disc protruded, I had to go to the hospital for acupuncture every day. It was really serious at that time. I could only rest in bed, and I needed help to walk and eat. I feel very embarrassed, very undignified, and at the same time disturbing the normal life of others." (Pianist 10)

Pianist 5 discussed the psychological stress she faced after suffering from PRMD and her confusion about future career development:

"Especially when the shoulder inflammation attacks, I feel very anxious and painful. I felt that I was unworthy of the expectations of my parents and piano teachers, and then gave myself a huge psychological pressure and burden. I feel a lot of remorse and remorse, and at the same time, I worry that if my illness worsens, I won't be able to become a Pianist in the future, and then I will fall into a confused state." (Pianist 5)

As student pianists who had not yet formally entered the professional world, the experience of PRMD significantly impacted participants’ future career plans. When discussing their career aspirations post-graduation, despite their passion for piano playing, some participants also leaned toward relinquishing the goal of becoming pianists due to the limitations imposed by their health conditions. As expressed by Pianist 7:

"I can not become a professional pianist. My current physical condition can not support me to be a professional pianist. I might be a teacher after graduation. I might teach children to play the piano or to be a music teacher in middle school." (Pianist 7)

#### 3.4.2 Positive meanings of PRMD

While suffering from PRMD was physically and mentally distressing, some participants also derived positive meanings from their experiences associated with PRMD. These include being more determined to become a professional pianist dream, valuing the opportunity to play the piano more, becoming more aware of health issues, and learning to accept negative experiences in life. Additionally, participants mentioned that they would empathize with pianists who had similar experiences and use their own experiences and methods to help others, transitioning from victims to helpers.

Despite the physical and mental pain experienced by some participants due to PRMD, their determination to become professional pianists in the future grew stronger. Pianist 2 emphasized that playing the piano was the meaning of his life. Pianist 8 expressed, “Not playing the piano would make me lose the love of life,” and Pianist 12 stated, “The piano is where my soul is.” Pianist 1 described:

"Piano is my friend, my confidant, my career and my hobby. So, no matter how bad my health is, I will play piano for the rest of my life. I'm going to be a professional pianist because I can not be anyone but a pianist." (Pianist 1)

Pianist 12 mentioned that she currently cherishes every opportunity to perform in public:

"I know I cannot play as much as I used to, so I cherish every opportunity to perform. Tomorrow and the accident do not know who will arrive first, and I cannot guarantee the chance to play all the time, so all I can do is treat each performance as if it were my last" (Pianist 12).

Pianist 5 started paying more attention to her health after suffering from PRMD:

"I pay special attention to my health now, watching my diet and rest. I will also give myself time to rest and not over-practice as much as I used to. I also try to be positive and control negative emotions and thoughts. My personality is much better than before, and I am no longer depressed and stubborn." (Pianist 5)

A member of Focus Group 1 mentioned that her experience with PRMD enabled her to accept other negative experiences in her life more readily:

"To be honest, this is one of the biggest bad experiences in my life right now, leaving me depressed and confused. But after that, I had setbacks and other bad experiences, and I found that I took it easy. My heart will not fall into the trough and depression, but first, think about the solution to the problem. That is what this experience has brought to my life. Experience itself is not good or bad; it is what we make of it."(Focus Group 2)

Pianist 4 mentioned that her experience with PRMD led to volunteer to help other injured pianists:

"I feel a sense of empathy now for pianists who have the same problems as me, especially for pianists who have gone through them after me and have not dealt with them yet. I will tell them about my treatments and some of my pain relief; I will also tell them about my experience to make them feel a little better." (Pianist 4)

## 4 Discussion

This study explores the lived experiences of Chinese tertiary student pianists concerning playing-related musculoskeletal disorder (PRMD), including their subjective thoughts, feelings, and the meaning of their PRMD-related experiences. Research indicates that student pianists’ perceptions associated with PRMD are multifaceted. The first and most immediate reaction participants felt was physical pain and discomfort. When recalling their initial experience with PRMD, participants were generally vague about the timing and course of the injury but could clearly remember how their bodies felt and responded. Additionally, the physical pain and discomfort caused by PRMD persisted, with participants reporting varying degrees of pain both at the piano and in daily life, alongside a risk that this physical pain would worsen over time.

These results support previous studies which found that musicians suffering from PRMD are not well cared for and treated due to the lack of currently recognized effective preventive and therapeutic measures, thus enduring long-term physical discomfort at work (Cruder et al., 2020). Moreover, the results illustrated that exposure to PRMD also induced changes in participants’ mindset and mood. After the injury, participants generally lost confidence in their playing ability, skills, and performance status, and felt uncertain about their career development post-graduation. Negative evaluations from others also affected their psychological state, leading to hurt self-esteem and even performance anxiety. Previous research has shown that musicians are susceptible to mental and emotional challenges, and that physical conditions, public evaluations, and poor performing experiences can result in psychological problems (Santos and Queirós, 2019; Sorensen et al., 2021).

Past studies confirmed that musicians experience negative emotional reactions after suffering PRMD, including sadness and depression over the injury, anxiety and confusion over career development, pain and discomfort over physical condition, and anger and despair over the inability to change the status quo (Sorensen et al., 2021). Howard’s (2004) research indicates that musicians’ experience post-injury is a cyclic process from loss to recovery, and musicians will eventually find a new balance and enter a new cycle. This study further corroborates Howard (2004), finding that participants’ emotions follow a similar cycle—from disbelief to acceptance. After experiencing feelings of disbelief, sadness, anxiety, hopelessness, and anger, participants eventually came to terms with the fact that they had suffered a PRMD, approaching the PRMD-related experience more peacefully and rationally.

Previous literature suggests that musicians belong to a profession with a strong sense of identity and high career satisfaction, and it is precisely because of this that they have a high sense of self-efficacy in playing the piano (Bandura, 1999), deriving happiness from their careers. Therefore, even when afflicted with PRMD, they continue to work (Park et al., 2007; School and Zosso, 2012). The study by Park et al. (2007) investigated the reasons why tertiary music students are willing to continue to perform despite the high risk of injury. They found that music students also identified as musicians and were not likely to abandon their music careers easily. This study supports this view. Participants believe that being a pianist brings them pride and happiness, viewing it as their social identity that reflects their abilities, strengths, dreams, and self-perception. However, participants also experienced a sense of uncertainty and conflict stemming from identity. As student pianists, their health problems are easily overlooked, and the public readily questions their performance ability. A study by McCready and Reid (2007) showed that music students also had experiences of having their music-playing career interrupted due to PRMD. The participants in this study had not yet formally started their careers, and suffering from PRMD also exposed them to career uncertainty. Finally, participants also faced an identity conflict between being a pianist and a patient, which caused a psychological burden, embarrassment, and low self-esteem. Past literature has also suggested that suffering from PRMD can reduce musicians’ job satisfaction, cause them to experience a career crisis, and evoke a feeling of “lost identity” (Sorensen et al., 2021).

The musicians’ descriptions suggest that their strategies for coping with injury are divided into two main categories: self-regulation and seeking assistance through social relationships. The concept of self-regulation comes from social cognitive theory (SCT), which proposes that self-regulation and self-efficacy can improve an individual’s sense of efficiency in behavior and performance (Bandura, 2002). Participants were more likely to self-regulate when choosing coping measures, including improving playing methods, adjusting lifestyle habits, and self-psychological adjustment. Because coping with PRMD is a long process, participants do not have the help and support of others at all times. Previous literature has also confirmed the role of participants in self-regulation, such as the use of scientific playing methods to relieve musculoskeletal stress and promote muscle self-repair (Ackermann et al., 2014; Pappa, 2019; Shanoff et al., 2019), improving the quality of life and exercise can alleviate the physical and mental pressure of musicians (Lee et al., 2012; Ajidahun et al., 2019). Past research has also confirmed the benefits of psychological practices for musicians managing stress and coping with PRMD (Kuo, 2012). This study further found that in addition to the emotional value provided by the professional treatment of psychotherapists and social relationships, self-psychological regulation conducted by participants, including positive mental suggestion, self-encouragement, and meditation, positively affected managing PRMD. Lazarus and Folkman's (1984) Transactional Model of Stress and Coping (TMSC) proposed two coping strategies: problem-focused and emotion-focused coping strategies. Problem-focused coping strategies include doing something to alleviate the pain people face, while emotion-focused coping strategies include reducing emotional pain and regulating emotional stress (Sittichai and Smith, 2018). The assistance provided by social relationships to participants also encompasses these two dimensions. The participants’ piano teachers and therapists provided problem-focused assistance. This included help in adjusting their playing habits and techniques and professional therapy. In addition, social relationships such as family, friends, teachers, classmates, and even listeners and employers can provide emotional support for participants, including companionship, encouragement, understanding, and tolerance. SCT proposes that social and family support is the most important support for individual behavior (Bandura, 2002), and past literature affirms the importance of trust, understanding, and a supportive work environment for musicians’ physical and mental health (Santos and Queirós, 2019). The study also demonstrated the benefits of positive social relationships on participants’ coping with PRMD and future career development. Even emotional support that does not help them solve actual problems can help them approach PRMD-related experiences more rationally and optimistically.

Participants described the impact and significance of their experiences with PRMD. The negative impact of PRMD on participants was inevitable, and the most obvious impact was reflected in their piano performance. Cruder et al. (2020) proposed that musicians suffering from PRMD would have difficulty controlling their performance, leading to career uncertainty. This study supports the perspective of Cruder et al. (2020) that due to physical pain and discomfort after injury, participants faced a decline in performance ability and unstable performance status, and the difficulty of playing pieces was reduced due to physical conditions. They suffered from PRMD because of playing the piano, and their performance was affected and limited by PRMD after the injury. The psychological state and daily life of the participants were also affected by PRMD. This study, like previous literature, found that the participants also faced psychological stress and negative emotions in addition to physical pain (Santos and Queirós, 2019), but the results of this study show that student pianists could adjust their psychology to a normal state through self-psychological adjustment and emotional support from social relationships. The results also demonstrate that the participants’ daily lives were affected by PRMD, and they may not be able to participate in daily labor and sports or even take care of their daily lives due to physical pain and discomfort. In the past, little literature mentioned the impact of PRMD on the daily life of musicians. Limited by the small sample size, this study may be unreliable, so more studies should focus on musicians’ lived experiences other than instrumental music.

Notably, this study found that participants’ experiences related to PRMD were also positive. Howard (2004) proposed that the experience of a musician’s injury is a cyclical process of loss to recovery, and musicians eventually find a new balance. In addition, there is little literature on the positive significance of PRMD for musicians because PRMD is a serious and potentially career-ending disease for musicians. However, the results of this study demonstrate that after long-term experience and coping with PRMD, participants became self-regulators, able to adjust their playing habits, living habits, and mental states, and re-establish high self-efficacy and high outcome expectations for piano performance (Bandura, 2002). While receiving substantial help and emotional support from social relations, they also sympathized and assisted other injured pianists, thus establishing good interpersonal relationships. Participants would also begin to value health issues and careers and learn from PRMD-related experiences the mindset and courage to face other negative experiences in their lives.

This study is conducted within the context of China, and in comparison with prior literature, the results are infused with East Asian cultural characteristics. Primarily, the results mirror the stringent school education and family education prevalent in East Asia (Bary and Chaffee, 1989). The participants often had ambitious parents and strict piano teachers, which forced them to practice excessively and increased the risk of PRMD. After injury, they also suffer from thoughts such as “failing to live up to the expectations of parents and teachers.” While existing literature indicates that oral drugs, injectable drugs, and surgery are prevalent treatments for musicians (Shanoff et al., 2019; Matei and Ginsborg, 2022), participants in this study exhibited a stronger preference for traditional Chinese medicine (TCM) treatment and expressed higher trust in TCM doctors. This contrasts with musicians in other countries who rarely consider TCM as their primary treatment choice. Finally, previous research indicates that injured student pianists, like professional pianists, are not inclined to easily abandon their playing careers (Pappa, 2019), but many participants in this study tend to give up playing and pursue other music-related careers when they suffer from more severe PRMD. The participants had the same love for playing the piano as other pianists, but they thought health was more important. They described this choice as “another avenue to fulfill the musical dream” in their own words.

## 5 Implications

This study employed a transcendental phenomenological approach to examine student pianists’ experiences in China’s higher education institutions concerning playing-related musculoskeletal disorder (PRMD), focusing on their thoughts and feelings. Currently, most of the literature related to PRMD consists of quantitative studies, with few investigating the lived experiences of musicians, and even fewer studies on student pianists. This study is based on the experiences of student pianists without the researchers’ interpretation of their experiences. Such an approach can lead one back to the essence of the matter, to the nature of the phenomena experienced by the student pianist (Moustakas, 1994).

This study utilizes Social Cognitive Theory (SCT) as a theoretical perspective (Bandura, 1986) and emphasizes the importance of enhancing student pianists’ self-efficacy and outcome expectations, as well as encouraging student pianists to become self-moderators. SCT has not been used in studies of musician health before. This study extends the theory and finds a feasible theoretical perspective for subsequent studies related to PRMD.

This study further underscores the importance of providing student pianists with quality music education, health education, and healthcare to cope with PRMD and sustain their playing careers. This study also provides a perspective for young student pianists in the field of musician-related healthcare, affording relevant personnel the opportunity to understand and value the experiences of student musicians related to PRMD and their thoughts and appeals to tailor injury coping strategies to them.

## 6 Limitations

This study’s limitation lies in relying on telephone interviews for some participants, specifically professional pianists, due to their time constraints resulting from treatments, concerts, and rehearsals. The necessity to accommodate their busy schedules might have impacted the depth and quality of information gathered compared to face-to-face interviews. Despite efforts to ensure informed consent and maintain anonymity, the phone call approach may have introduced potential limitations, such as a potential loss of non-verbal cues and a different level of engagement that could influence the richness of the data obtained. In addition, this study is the first to investigate the experiences of Chinese musicians related to PRMD. Although the study subjects are a homogeneous group of tertiary student pianists, the findings cannot be generalized to the broader group of Chinese musicians. The research related to PRMD in China is still in its infancy, with little relevant literature and a lack of influential studies, leading to long-term neglect of musicians’ health issues, including their experiences related to PRMD. This study can serve as a preliminary study in this field in China, inspiring more people to pay attention to musicians’ health issues and experiences and establishing a supportive work environment.

## 7 Conclusion

This study investigated the experiences of Chinese tertiary student pianists related to playing-related musculoskeletal disorder (PRMD), providing a perspective for young Eastern Asian pianists in the field of performing arts medicine. Simultaneously, as the first study in China to explore the experiences of musicians suffering from PRMD, this study can serve as a preliminary study to inspire more people to pay attention to injured musicians. Future research needs to continue to explore the relationship between musicians’ lived experiences and PRMD, and how musicians’ experiences can be used to develop prevention and treatment strategies applicable to them. Future research also needs to focus on student musicians, including pianists, whose situations and experiences differ from those of professional musicians, yet the impact of suffering from PRMD on their physical and mental health and future careers is significant. Additionally, it is suggested that Chinese researchers employ quantitative studies to determine the prevalence and risk factors of PRMD in China, to gain a more comprehensive understanding of the basic situation of PRMD among Chinese musicians. Researchers can also focus on the health problems of players of traditional Chinese instruments such as erhu, guzheng, pipa, and guqin to enrich the existing literature.

## Data availability statement

The original contributions presented in the study are included in the article/Supplementary material, further inquiries can be directed to the corresponding author.

## Ethics statement

The studies involving humans were approved by Ethics Committee for Research involving Human Subjects Universiti Putra Malaysia. The studies were conducted in accordance with the local legislation and institutional requirements. The participants provided their written informed consent to participate in this study.

## Author contributions

MX: Data curation, Methodology, Writing – original draft, Writing – review & editing. AM: Methodology, Supervision, Writing – review & editing. IS: Supervision, Writing – review & editing.
