# Supplementary material for: Subjective experiences of tertiary student pianists with playing-related musculoskeletal disorder: a transcendental phenomenological analysis
Source: Front Psychol. 2024 Apr 23;15:1303046. doi: 10.3389/fpsyg.2024.1303046 (PMC11075168; doi:10.3389/fpsyg.2024.1303046)
Supplement: Supplementary file 1 [file Table_1.DOCX]

**Appendix 1 Interview Guide.**

| Interview Guide |
| --- |
| 1. Please tell me how your PRMD happened.   - When did it happen? - How did it happen?   2. What are the specific manifestations and symptoms of your PRMD?  3. What are the risk factors that cause you to suffer from PRMD?  4. Do you have any effective methods to prevent and alleviate PRMD?  5. What PRMD related treatments have you ever received?   - Who gives you treated? - Why did you choose this treatment? - Are these treatments helping your condition?  1. How do you view your identity as a pianist or an injured pianist? 2. What happens to your body after PRMD (how does your body feel)? 3. What is your psychological feeling after suffering from PRMD? 4. What influences, difficulties, changes or gains have PRMD-related experiences brought to you? 5. Please tell me about your other important PRMD-related experiences, thoughts or perspectives not mentioned in this interview. |
